# Transcriptomic analysis reveals impaired tight junction and upregulated IL-17 signaling in induced abnormal keratinization area of rumen

**DOI:** 10.1093/jas/skaf352

**Published:** 2025-10-14

**Authors:** Tianxi Zhang, Zhiyuan Ma, Fei Li, Fenja Klevenhusen, Long Guo, Li Wang, Zhian Zhang, Shuai Jiao, Fang Luo, Tao Guo, Xinji Wang, Kaidong Li, Baocang Liu

**Affiliations:** State Key Laboratory of Herbage Improvement and Grassland Agro-ecosystems, College of Pastoral Agriculture Science and Technology, Lanzhou University, Lanzhou, 730020, China; State Key Laboratory of Herbage Improvement and Grassland Agro-ecosystems, College of Pastoral Agriculture Science and Technology, Lanzhou University, Lanzhou, 730020, China; State Key Laboratory of Herbage Improvement and Grassland Agro-ecosystems, College of Pastoral Agriculture Science and Technology, Lanzhou University, Lanzhou, 730020, China; Section of Environmentally Sustainable Animal Nutrition, Faculty of Organic Agricultural Sciences, University of Kassel, Witzenhausen, 37213, Germany; State Key Laboratory of Herbage Improvement and Grassland Agro-ecosystems, College of Pastoral Agriculture Science and Technology, Lanzhou University, Lanzhou, 730020, China; State Key Laboratory of Herbage Improvement and Grassland Agro-ecosystems, College of Pastoral Agriculture Science and Technology, Lanzhou University, Lanzhou, 730020, China; State Key Laboratory of Herbage Improvement and Grassland Agro-ecosystems, College of Pastoral Agriculture Science and Technology, Lanzhou University, Lanzhou, 730020, China; State Key Laboratory of Herbage Improvement and Grassland Agro-ecosystems, College of Pastoral Agriculture Science and Technology, Lanzhou University, Lanzhou, 730020, China; State Key Laboratory of Herbage Improvement and Grassland Agro-ecosystems, College of Pastoral Agriculture Science and Technology, Lanzhou University, Lanzhou, 730020, China; State Key Laboratory of Herbage Improvement and Grassland Agro-ecosystems, College of Pastoral Agriculture Science and Technology, Lanzhou University, Lanzhou, 730020, China; Animal Husbandry and Veterinary Station, Minqin, 733399, China; Agricultural and Rural Bureau Zhongxing Town Animal Husbandry and Veterinary Station, Minqin, 733399, China; Changji Feed Co., Ltd of Xinjiang TaiKun Group, 831100, China

**Keywords:** pH, rumen, abnormal keratinization, transcriptome

## Abstract

High-grain diets can reduce rumen pH, which may damage the process of keratinization of the ruminal stratified squamous epithelium, leading to areas of abnormal keratinization (AK). However, the comprehension of molecular biological processes leading to the development of an AK area in the rumen is limited. A total of 48 wethers (2-month-old) were fed a diet containing 48% barley and 24% starch (DM basis) to induce AK development. Rumen fluid samples were collected via an oral stomach tube at 0, 2.5, and 6 h post-morning feeding on three consecutive days (d 58, 59, and 60) to measure pH. Following a 63-day feeding period, all lambs were slaughtered, and based on macroscopic pathological observation of the rumen, they were retrospectively classified into two categories of individuals: individuals with normal rumen (INR; *n* = 38) and individuals with abnormal keratinization rumen (IAKR; *n* = 10). Tissue samples from the AK and adjacent morphologically normal (MN) areas of IAKR were analyzed histologically and via RNA sequencing and quantitative real-time PCR (qRT-PCR). Statistical analysis used Shapiro-Wilk tests for histological and qRT-PCR data and linear mixed models for pH comparisons. Key genes and pathways were identified through Differential Expression Analysis (DEA), Weighted Gene Co-expression Network Analysis (WGCNA), and Gene Set Enrichment Analysis (GSEA). Compared to the INR, the IAKR had a lower rumen pH at 2.5 h post-morning feeding (5.352 vs 6.035, *P *< 0.01). Histological examination showed significant reductions in papillae length, width, and stratum corneum thickness in the AK area compared to the MN area (*P *< 0.01). Transcriptomic analysis revealed the gene expression downregulation of ACTR2, which play a role in tight junctions; Downregulation of SLC38A2, a gene involved in the protein digestion and absorption pathway, was also observed; The upregulation of MMP9 was observed in the IL-17 signaling pathway, contributing to tissue damage; The expression of ITGB7 was also upregulated, which intensified the local immune response. The expression patterns of these genes were further confirmed by qRT-PCR analysis. In conclusion, the current study revealed molecular processes involved in the disruption of intercellular connections and structural support in tissue cells and impaired ability of cells to absorb nutrients and capture signaling molecules within these AK areas caused by high grain feeding.

## Teaser

This study reveals the potential pathogenesis and transcriptomic features of the abnormal keratinization area of the rumen and provides a reference for research on rumen health in ruminants.

## Introduction

The rumen provides about 70% of the energy needs of ruminants through efficient microbial fermentation and nutrient absorption (Krehbiel, 2014). The health and proper functioning of the rumen are critical to the efficient nutrient utilization and metabolic health of ruminants (Baaske et al., 2020). Improper feeding management, changes in diet composition and toxic substances in the diet can all lead to abnormal rumen function or even substantial injury (Kovács et al., 2020). For example, in ruminant production, feeding strategies that aim to enhance productivity often involve high-grain diets, which can lead to an increased formation of volatile fatty acid (VFA) and lactic acid in the rumen, causing a decrease in pH, which may affect keratinization of the rumen epithelial cells (Zhang et al., 2024). High levels of potentially toxic substances in the diet and rumen contents, such as tannins, mycotoxins, and lipopolysaccharides, may also damage the rumen epithelium and lead to keratinization abnormalities (Broom, 2015).

In the digestive tract of ruminants, the stratum corneum is a critical protective structure in the rumen. It forms the outermost layer of the rumen epithelium and is composed of corneocytes derived from proliferative keratinocytes in the underlying stratum basale (Zhang et al., 2024). During keratinocyte differentiation, these cells undergo morphological flattening and denucleation, subsequently integrating with lipids to form a robust protective structure (Gonzales and Fuchs, 2017). Damage to the rumen stratum corneum compromises its barrier function and impairs digestive and absorptive capacity (Nasrollahi, 2023). Abnormal keratinization (AK; including parakeratosis and dyskeratosis) in the rumen, which can be directly caused by overly low pH as seen in subacute ruminal acidosis (SARA), is difficult to detect ante-mortem (Li et al., 2019; Fayazi et al., 2020). However, the molecular basis underlying the histopathological features of AK in ruminal tissue remains poorly understood.

To explore the underlying molecular changes associated with rumen AK, particularly focusing on epithelial barrier function, inflammatory responses, and nutrient absorption pathways, we fed lambs a barley-rich diet with high SARA-inducing potential to develop AK in the rumen. While previous studies have described morphological changes associated with SARA-induced rumen damage, the detailed molecular mechanisms governing AK development remain poorly understood. We hypothesized that this dietary approach would induce rumen AK and that transcriptomic analysis would reveal specific molecular pathways associated with epithelial barrier dysfunction and inflammatory responses in affected tissue areas. We compared rumen pH before and after feeding between individuals with normal rumen (INR) and individuals with abnormal keratinization of the rumen (IAKR), as well as the gene expression at transcript levels between the AK area and the adjacent morphologically normal (MN) area of the rumen. This study aimed to: (1) characterize the transcriptomic profile of AK areas in rumen epithelium; (2) identify key molecular pathways associated with epithelial barrier dysfunction; and (3) elucidate the relationship between inflammatory responses and structural damage in affected tissues.

## Methods

### Experimental design, animals, and diets

The experimental protocol was approved by the Animal Ethics Committee of Lanzhou University (Lanzhou, China), and humane animal care and handling procedures were implemented throughout the experiment (CPAST-MA-2023-1).

This study was a self-controlled trial comparing the pathological histomorphology and transcriptome composition between areas of abnormal keratinization (AK) and adjacent morphologically normal (MN) areas in the rumen of individuals with abnormal keratinization of the rumen. To induce abnormal keratinization rumen, a total of 48 Hu wethers (2-month-old, 22.8 ± 0.233 kg body weight; mean ± SD) were fed the high SARA-inducing potential diet, which contained 48% barley and 24% starch (DM basis; Table 1). Following slaughter, animals were retrospectively classified into two categories of individuals based on rumen examination: (1) individuals with normal rumen (INR)—animals showing no abnormal keratinization, and (2) individuals with abnormal keratinization rumen (IAKR)—animals displaying abnormal keratinization in the ventral sac. Subsequently, AK and MN rumen tissue samples were obtained from the IAKR for analysis. Lambs were fed daily at 07:00 and 18:00 h in individual pens with *ad libitum* access to feed (average daily feed intake, INR = 1.627 ± 0.067 kg; IAKR = 1.582 ± 0.067 kg), allowing a 10% residual, and they have free access to water. On day 58, 59, and 60 of the experiment, rumen fluid samples were collected at 0, 2.5, and 6 h after the morning feeding. Rumen fluid samples (about 100 mL) were collected using an oro-esophageal tube (Kelibo Animal Husbandry Technology Co. Ltd, Wuhan, China), discarding the first 20 mL to avoid saliva contamination (Keefe and Ogilvie, 1997; Duffield et al., 2004; Ramos-Morales et al., 2014). The collected rumen fluid was immediately measured for pH using a portable pH meter (Starter 300; Ohaus Instruments Co. Ltd, Shanghai, China). After slaughter, animals were retrospectively classified into two groups based on rumen examination: (1) individuals with normal rumen (INR)—animals showing no abnormal keratinization, and (2) individuals with abnormal keratinization rumen (IAKR)—animals displaying abnormal keratinization in the ventral sac.

**Table 1.** Ingredients and chemical composition of diets (% DM basis)

Items	Basal diet
**Ingredients**	
**Barley straw**	15.00
**Barley**	48.00
**Corn bran**	15.50
**Cottonseed meal**	6.00
**Soybean meal**	8.00
**Molasses**	4.00
**Sodium bicarbonate**	0.60
**Limestone**	1.20
**Salt**	0.70
**Premix^1^**	1.00
**Nutrient content**	
**DM**	91.81
**CP**	13.69
**NDF**	31.25
**ADF**	14.28
**Starch**	24.02
**Ca**	0.83
**P**	0.31
**Metabolizable energy (MJ/kg)**	9.57

1 The premix provided the following per kg of diet: Fe, 25 mg; Mn, 40 mg; Zn, 40 mg; Cu, 8 mg; I, 0.3 mg; Se, 0.2 mg; Co, 0.1 mg; VA, 940 IU; VD, 111 IU; VE, 20 IU.

### Rumen tissue sample collection

On day 63 of the experiment, all lambs were electrically stunned after a 12-h fasting period and euthanized by exsanguination through severing both the carotid artery and jugular vein. After isolating the rumen and clearing its contents, pathological morphology was observed macroscopically to identify IAKR. The AK of the rumen were identified based on the following criteria according to Luna-Méndez et al (2020): (1) Observing an unusually thick or hyperplastic stratum corneum on the rumen epithelium; (2) Noting rough, dry, or irregular surfaces on the ruminal lining; (3) Identifying abnormal coloration, such as yellowing or whitish patches, often indicative of keratin buildup; (4) Detecting area where rumen papillae are reduced, flattened, or completely absent. In this study, AK areas were identified primarily based on criteria (3) and (4). Abnormal keratinization areas were consistently localized on the ventral ruminal sac at the floor permanently in contact with digesta; no macroscopic lesions were observed on the dorsal, caudo-dorsal, or caudo-ventral sacs. Among the 48 lambs, only 10 individuals (20.8%) developed visible AK areas, which were designated as IAKR. The remaining 38 lambs showed normal rumen epithelium and were classified as INR. From each of the 10 IAKR animals, four tissue subsamples were collected from the AK area and four additional subsamples from the adjacent MN area within the same animal. The samples were immediately rinsed in cold phosphate-buffered saline (PBS) to clean the attached rumen contents. Three rumen mucosa samples separated from the rumen sample by blunt dissection were snap-frozen in liquid nitrogen for RNA extraction. Rumen tissue samples with both mucosa and muscle layers were fixed in 4% paraformaldehyde solution (Servicebio Biotechnology Co. Ltd, Wuhan, China) for histological examination.

### Histological examination

The fixed rumen tissue samples were dehydrated through serial concentrations of anhydrous ethanol (50%, 70%, 80%, 90%, and 100%), followed by two rounds of xylene clearing, and then infiltrated and embedded in paraffin. Hematoxylin-Eosin staining was performed according to a standard method (Li et al., 2018). The tissue sections were observed using a Pannoramic digital slide scanner (3DHISTECH, Hungary). The Caseviewer image analysis software (version 2.4) was employed, and the measurement tools “Draw an open polygon” and “Linear measurement annotation” were selected from the Annotation section. In this experiment, three parameters were measured in the rumen: papilla height, papilla width, and stratum corneum thickness. Measurements were performed at 100× magnification for papillae length and width, and at 400× magnification for stratum corneum thickness determinations. At least five microscopic fields of the view were selected from each sample, and the length and width of the rumen papillae were measured in each field. The stratum corneum thickness was measured at a minimum of 20 locations for each sample. The arithmetic mean of each sample was calculated, with different samples treated as independent observations.

### Rumen RNA sequencing and bioinformatic analysis

Total RNA from rumen mucosa samples was extracted using Trizol (Tsingke Biotechnology Co. Ltd, Xian, China) according to the manual. Subsequently, total RNA was characterized and quantified using a NanoDrop and Agilent 2,100 Bioanalyzer (Thermo Fisher Scientific, MA, United States).

Oligo (dT)-linked magnetic beads were used to enrich mRNA. The enriched mRNA was fragmented using an RNA-fragmentation kit (Thermo Fisher Scientific, Shanghai, China). First-strand cDNA was then generated using random hexamer-triggered reverse transcription, followed by the synthesis of second-strand cDNA. After which, A-Tailing Mix and RNA Index Adapters (New England Biolabs, USA) were inserted by incubation to conclude the repair. The cDNA fragment obtained in the previous step was amplified by PCR and the product was purified by Ampure XP Beads and then dissolved in EB solution. The product was validated for quality control on an Agilent Technologies 2,100 Bioanalyzer (Agilent Technologies, Germany). The obtained cDNA was subjected to paired-end sequencing on an Illumina Novaseq PE150 platform (Illumina, USA). The raw reads were processed using Trimmomatic (version 0.39) (Bolger et al., 2014) for quality control. Specifically, reads with more than 10% unknown bases, reads consisting entirely of base A, adapted, and reads with more than 50% of bases having a quality score (Q) ≤ 20 were excluded, resulting in clean reads. The rRNA reads were removed from the clean reads by aligning to the rRNA database (Cole et al., 2014) using Bowtie2 (v2.2.8) (Langmead and Salzberg, 2012). Paired-end mRNA reads were then mapped to the lamb genome (*Ovis aries*, Oar_v3.1, https://ftp.ensembl.org/pub/release-110/fasta/ovis_aries/dna/Ovis_aries.Oar_v3.1.dna.toplevel.fa.gz) using HISAT (Zhang et al., 2021), and the mapped reads were assembled into transcripts using StringTie (version 1.3.1) (Pertea et al., 2015). Gene expression levels were quantified by calculating the fragments per kilobase of exon model per million mapped fragments (FPKM). Kyoto Encyclopedia of Genes and Genomes (KEGG) pathway annotation of the differentially expressed genes (DEGs) was performed.

Weighted Gene Co-expression Network Analysis (WGCNA) was used to investigate the relationship between abnormal morphological indicators of the rumen and transcriptional changes. Genes with an average FPKM > 2 across all rumen samples were selected and imported into the WGCNA R package (Langfelder and Horvath, 2008).

Gene Set Enrichment Analysis (GSEA) was employed to assess the overall expression changes of KEGG gene sets in the MN and AK area of the rumen of IAKR (https://www.gsea-msigdb.org/gsea/index.jsp). This approach identified gene sets exhibiting coordinated expression changes from the expression matrix of all genes. The R package aPEAR (Advanced Pathway Enrichment Analysis Representation) was used to cluster gseKEGG pathways based on their similarity and represent the results as an enrichment network (Kerseviciute and Gordevicius, 2023). Gene interactions were identified through the String database (https://string-db.org) and visualized using Cytoscape (version 3.10.3) to explore the relationships between genes.

### qRT-PCR analysis of genes in rumen epithelium

The extracted total RNA of the rumen mucosa was used for reverse transcription using a SynScript III RT SuperMix reagent kit with gDNA Remover (Tsingke Biotechnology Co. Ltd, Xi’an, China). Quantitative real time PCR (qRT-PCR) was performed to determine gene expression using ArtiCanATM SYBR qPCR Mix (Tsingke Biotechnology Co. Ltd, Xi’an, China) at a LightCycler 480 II Instrument (Roche, Basel, Switzerland). Relative expression of all target genes was determined and normalized to the reference genes (GAPDH) using the 2^-ΔΔCt^ method. GAPDH expression stability was validated using the geNorm algorithm (CV < 5% across all samples), confirming its suitability as a reference gene under our experimental conditions. Future studies would benefit from employing multiple validated reference genes for enhanced normalization robustness. Primers were synthesized by Tsingke Biotechnology Co., Ltd (Xi’an, China; Supplementary Table S1). Transcription efficiency was assessed using Agilent 2100 Bioanalyzer, with all samples showing RNA integrity numbers (RIN) > 7.0. Primer specificity was validated through melting curve analysis and gel electrophoresis of PCR products. Amplification efficiencies ranged from 95-105% for all primer pairs.

### Statistics

The sample size of 48 lambs was determined based on previous literature in ruminant nutrition research examining subacute ruminal acidosis and epithelial morphology changes. Given the expected incidence rate of abnormal keratinization (approximately 20–25% based on Luna-Méndez et al. [2020]), we calculated that 48 animals would provide sufficient statistical power (> 80%) to detect meaningful differences in rumen pH and morphological parameters between groups, assuming an effect size of 0.8, α = 0.05, and accounting for potential animal variability. This calculation anticipated identifying 8–12 animals with abnormal keratinization, which aligns with our actual findings (10 IAKR animals).

All statistics were performed with SPSS 24.0. Papillae length and width, stratum corneum thickness, and qRT-PCR gene expression levels of rumen tissue were assessed for normality using the Shapiro-Wilk test, confirming that they followed a normal distribution. Rumen pH between IAKR and INR was analyzed using the linear mixed model, with group, sampling time, and their interactions as fixed factors, animal as a random factor, and sampling time also been set as a repeated measurement with AR (1) correlation matrix as covariance structure. The AR (1) is selected based on the minimum Bayesian Information Criterion (BIC) value obtained through model fitting. When the interaction between group and time points was detected, a reduced linear mixed model, including group as a fixed factor and animal as a random factor, was used to analyze the differences between groups at each time point. Variables measured at a single time point, paired sample *t*-tests were conducted to evaluate the significance of rumen pH differences between IAKR and INR. To exclude transcriptomic genes with low abundance, we applied a filter of “FPKM > 1 and at least 50% appearance in all samples” to define expressed genes, which were then used for downstream analyses. Differentially expressed genes between the MN and AK area of the rumen of IAKR were identified using DESeq2 (Love et al., 2014). A gene co-expression network was constructed using the soft-thresholding method based on Pearson correlation, with a power value of 13. Gene modules were defined with the following parameters: a minimum of 100 genes per module, a maximum of 30 modules, and a reassignment threshold of 0.1. The relationships between the gene modules and phenotypes were assessed by Pearson correlation analysis. Differences were considered significant at *P*-value < 0.05. Differentially expressed genes were selected based on *P *< 0.05 and an absolute log2 (fold change) value >1. Weighted Gene Co-expression Network Analysis gene modules with |r| > 0.50 and *P *< 0.01 were considered significantly associated with the phenotype. Gene sets for GSEA were selected according to the specified condition of | Normalized Enrichment Score (NES) | > 1, nominal *P *< 0.05, False Discovery Rate (FDR) q-value < 0.25. The Benjamini-Hochberg correction was applied to adjust the *P*-values.

## Results

### Rumen pH and incidence of abnormal keratinization

**Figure 1.**
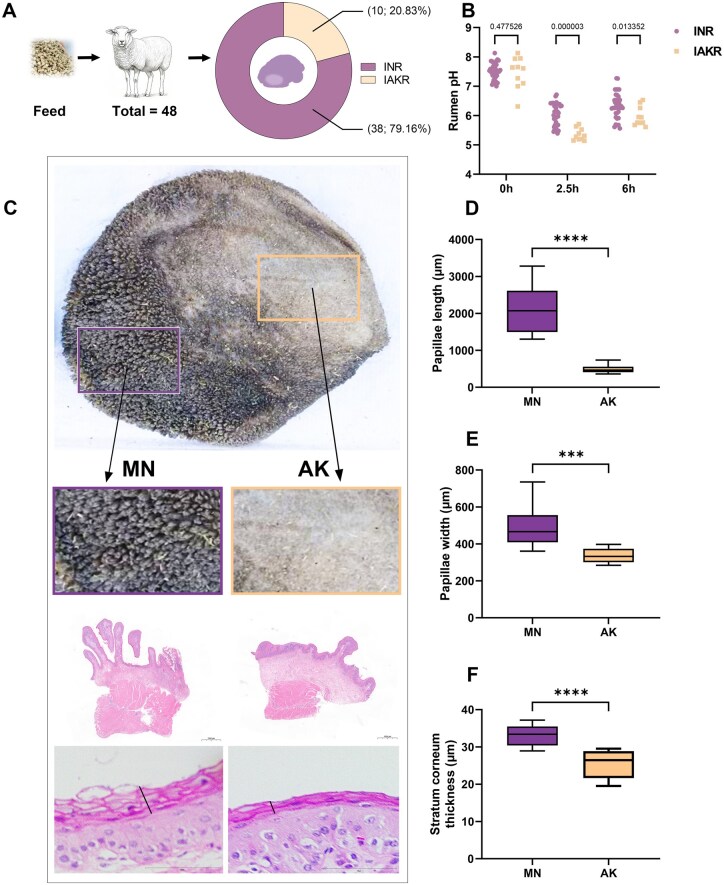
Barley-rich diet reduced rumen pH, leading to abnormal keratinization in the rumen. (A) Summary of the experimental procedure and the prevalence of individuals with abnormal keratinization of the ventral rumen epithelium; (B) pH levels of rumen fluid in lambs with INR and IAKR; (C) Morphological changes in the rumen papillae and the stratum corneum of the ventral rumen epithelium; (D–F) Measurements of the length, width of the rumen papillae and stratum corneum thickness. INR: individuals with normal rumen; IAKR: individuals with abnormal keratinization rumen; AK: abnormal keratinization area of the rumen; MN: morphologically normal area of the rumen (collected from tissue adjacent to the AK area). ***indicates a *P* < 0.001, and ****indicates a *P* < 0.0001.

A total of 10 IAKR were identified, and the remaining 38 lambs were identified as INR after slaughtering (Figure 1A). The linear mixed model analysis revealed an interaction between IAKR, INR, and sampling time (significant group × time interaction effect). Group differences at each time point were assessed with group as a fixed factor. The results showed that the rumen pH of the IAKR was lower (*P *< 0.01) at both 2.5 h and 6 h post-morning feeding, but was similar before morning feeding compared to the INR group (Figure 1B).

### Histological changes in rumen epithelium

Compared to the MN, the clinical abnormalities in the AK were characterized by localized white plaques in the ventral sac of the rumen, loosened epithelial tissue, reduced tissue integrity, and a visibly evident decrease in rumen papilla length (Figure 1C). Microscopic examination revealed that, compared to the MN, the length (Figure 1D), width (Figure 1E), and stratum corneum thickness (Figure 1F) of the rumen papillae were reduced (*P *< 0.01) in the AK.

### Differential gene expression analysis

**Figure 2.**
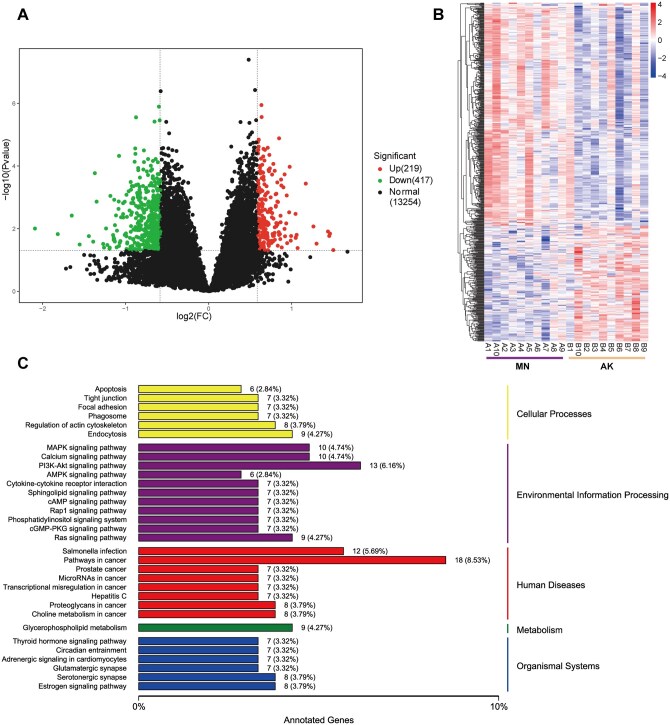
RNA-seq differential analysis reveals significant alterations in the expression patterns of abnormal keratinization area in rumen. (A) Differentially expressed genes between MN and AK; (B) Heatmap of gene expression in rumen tissues; (C) Kyoto encyclopedia of genes and genomes (KEGG) annotation and pathway type classification of downregulated differential expressed genes in AK. MN: morphologically normal area of the rumen; AK: abnormal keratinization area of the rumen.

A total of 636 DEGs were detected through transcriptomic analysis, with 219 upregulated and 417 downregulated in AK (*P *< 0.05 and an absolute log2 (fold change) value >1; Figure 2A; Supplementary Table S2). Hierarchical clustering analysis of the annotated genes revealed distinct gene expression patterns between MN and AK (Figure 2B). The KEGG category annotation of the DEGs shows the main downregulated pathways including cellular processes, environmental information processing, and metabolic pathways related to human diseases and organismal systems. Notably, pathways including tight junction and focal adhesion, were downregulated in the cellular processes category. Additionally, the endocytosis pathway was also downregulated (*P *< 0.05; Figure 2C).

### Weighted gene co-expression network analysis

**Figure 3.**
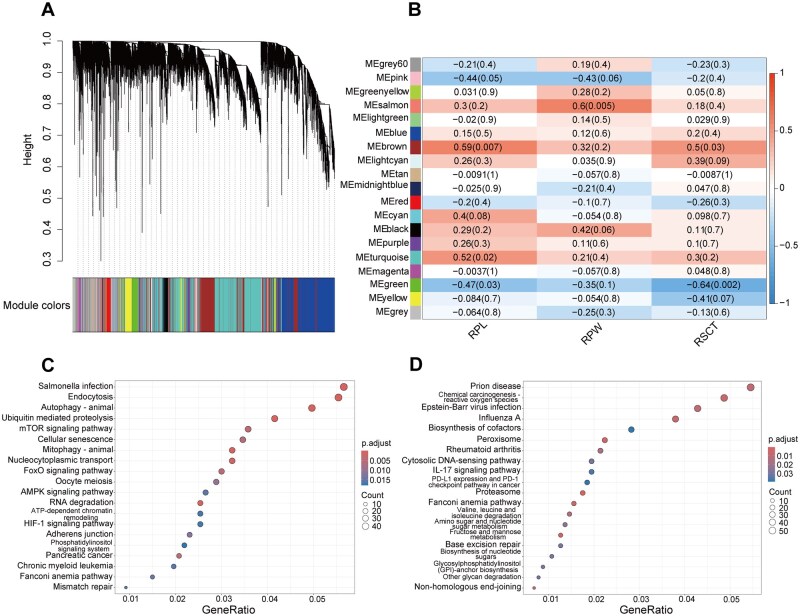
Weighted gene co-expression network analysis (WGCNA) reveals associations between rumen morphological indicators and gene modules. (A) Gene co-expression network modules; (B) Correlation heatmap between rumen morphological indicators and modules; (C) KEGG enrichment analysis of genes in the MEbrown module; (D) KEGG enrichment analysis of genes in the MEgreen module. RPL: rumen papillae length; RPW: rumen papillae width; RSCT: Rumen stratum corneum thickness.

To further explore the relationship between transcriptomic changes and morphological abnormalities in rumen tissues, we performed WGCNA on both the morphological data and transcriptomic results, generating 19 co-expression modules (Figure 3A). Among these, the MEbrown module showed a significant positive correlation with rumen papilla length, while the MEgreen module exhibited a significant negative correlation with the thickness of the rumen stratum corneum (*P *< 0.05; Figure 3B; Supplementary Tables S3–S5). Based on these findings, genes from these 2 modules were selected for KEGG enrichment analysis. The MEbrown module was enriched for the endocytosis pathway, as well as adhesion junction related to cell connections (*P *< 0.05; Figure 3C). Additionally, the MEgreen module showed significant enrichment of the IL-17 signaling pathway, which is involved in inflammation (*P *< 0.05; Figure 3D).

### Gene set enrichment analysis

**Figure 4.**
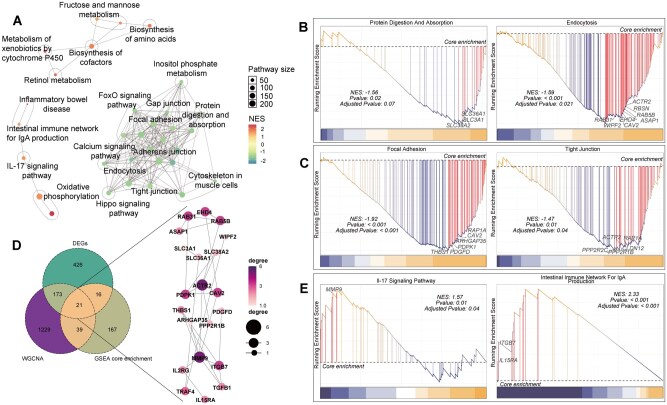
Gene set enrichment analysis (GSEA) evaluates the overall expression changes of KEGG gene sets between MN and AK samples. (A) gseKEGG pathway similarity clustering network; (B) Gene ranking for the protein digestion and absorption and endocytosis pathways; (C) Gene ranking for the focal adhesion and tight junction pathways; (D) Network interactions of candidate genes identified by three distinct methods (DEGs∩WGCNA module genes∩GSEA core enrichment); (E) Gene ranking for the IL -17 signaling pathway and intestinal immune network for IgA production pathways. MN: morphologically normal area of the rumen; AK: abnormal keratinization area of the rumen.

Through the gseKEGG network analysis of all expressed genes, we found that compared to MN, 21 major pathways were significantly downregulated in the AK, while 11 major pathways were upregulated. The downregulated pathways included tight junction, focal adhesion, adhesion junction, protein digestion and absorption, and endocytosis. The upregulated pathways included IL-17 signaling pathway and intestinal immune network for IgA production (Figure 4A; Supplementary Table S6). Based on the functional classification of differentially expressed genes and the results from WGCNA module gene enrichment analysis, we categorized protein digestion and absorption, and endocytosis as key pathways for digestion and absorption (Figure 4B; Supplementary Table S6), focal adhesion and tight junction as key pathways for maintaining tissue and cell structure (Figure 4C; Supplementary Table S6), and IL-17 signaling pathway and intestinal immune network for IgA production as immune and inflammation-related pathways (Figure 4E; Supplementary Table S6). We also analyzed the ranking of all genes within six categorized pathways, and identified the intersection genes between the DEGs and the highly correlated genes from the WGCNA strong modules in the core enriched genes of each pathway (Figure 4B–E, Supplementary Table S7). We further constructed an interaction network of these DEGs and found that *ACTR2* (actin related protein 2), *ITGB7* (integrin beta 7), *MMP9* (matrix metallopeptidase 9), *SLC38A2* (solute carrier family 38 member 2), and *RAB5B* (ras-related protein Rab-5B), interacted with each other (Figure 4D).

### qRT-PCR validation of key genes

**Figure 5.**
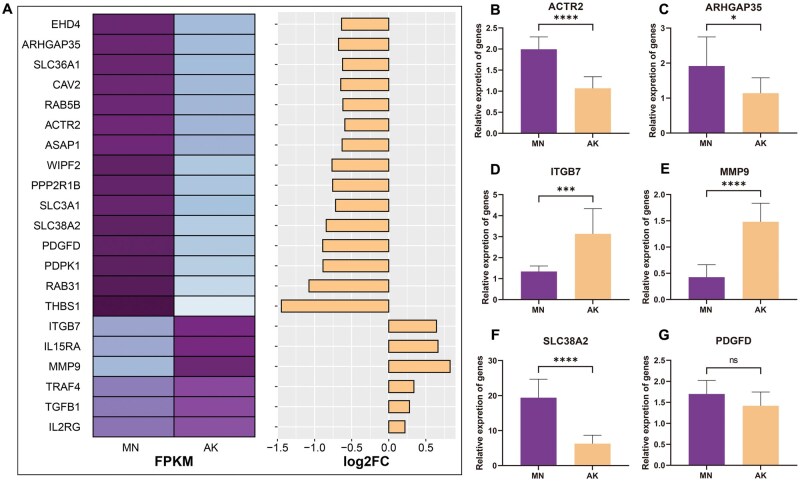
qRT-PCR validation of transcriptome data reliability. (A) FPKM and log2FC values for candidate genes from the transcriptome data; (B–G) q-PCR verification of expression levels for related genes. MN: morphologically normal area of the rumen; AK: abnormal keratinization area of the rumen. *indicates a *P *< 0.05, ***indicates a *P *< 0.001, and ****indicates a *P *< 0.0001. ns indicates that the difference is not statistically significant *P *≥ 0.05.

Finally, we performed qRT-PCR on the six genes obtained from the intersection of DEA, WGCNA, and GSEA to verify the reliability of the transcriptomic data. Compared to MN, the expression levels of *ACTR2*, *ARHGAP35* (Rho GTPase activating protein 35), and *SLC38A2* were significantly lower in AK, while the expression levels of *ITGB7* and *MMP9* were greater (*P *< 0.01) in AK. No difference in *PDGFD* (platelet derived growth factor) expression was observed (*P *≥ 0.05). The qRT-PCR results showed that the gene expression trends in rumen tissue were consistent with the transcriptomic data (Figure 5).

## Discussion

High-grain diets, while promoting faster animal growth, also lead to rapid fermentation in the rumen, which can result in excessive VFA concentrations and increase the risk of SARA (Plaizier et al., 2008). One of the most significant issues associated with SARA is the rumen tissue lesions (Zhang et al., 2017). Steele et al. (2011) demonstrated that high-grain diets caused rumen papillae to exhibit compromised cellular junctions and extensive sloughing of the stratum corneum in the rumen ventral sac, which may be related to the fact that the rumen ventral sac has prolonged contact with rumen fluid and is more severely affected by low pH compared to other parts of the rumen. In the present study 10 IAKR were observed among the 48 experimental animals, accounting for 20.83% of the total, indicating a notably high incidence under the dietary condition of a barley-rich diet. Luna-Méndez et al., (2020) reported that feeding a high-grain diet to finishing cattle resulted in 23% of cases showed AK of the rumen, an incidence close to that of the current study. Morphologically, AK of the rumen is characterized by reductions in the length, width, and stratum corneum thickness of the rumen papillae, signifying substantial alterations to the epithelial structure (Zhang et al., 2019). Induced SARA cattle with rumen pH to <5.8 for 5 to 6 h/d (Zebeli et al., 2012) or to < 5.6 for 3 h/d (Gozho et al., 2005) are strongly correlated with pathological changes in rumen epithelial morphology and histology, as also observed in the present study. Besides, we observed whitening of the rumen epithelium at the bottom of the ventral sac. When the rumen epithelium is impaired or even detached, the newly formed mucosa shows incomplete keratinization and reduced pigmentation compared to the mature mucosa which are typical symptoms of AK of the rumen (Steele et al., 2009; Luna-Méndez et al., 2020). While oro-esophageal sampling may introduce some variability due to potential saliva contamination, our methodology incorporated established protocols including adequate insertion depth, discarding initial samples, and multiple time-point sampling to minimize these effects (Keefe and Ogilvie, 1997; Garrett et al., 1999; Duffield et al., 2004; Ramos-Morales et al., 2014). Importantly, saliva contamination typically elevates pH measurements, making our identification of animals with lower pH values conservative and reliable indicators of ruminal acidosis.

The rumen stratum corneum serves as a critical physical barrier protecting the underlying epithelium (Baldwin and Connor, 2017). In abnormal keratinization conditions, this barrier function becomes compromised, making it easier for microbes to invade the mucosal layers (Fu et al., 2022). Tight junctions play a fundamental role in maintaining this barrier integrity by linking adjacent epithelial cells through cytoskeletal connections, thereby providing mechanical stability and contributing to overall tissue resilience (Zihni et al., 2016). Focal adhesions are dynamic, multi-protein complexes that physically connect the cellular cytoskeleton to the extracellular matrix, serving as mechanosensitive structures that transmit forces between the cell interior and exterior environment (Bauer et al., 2019). These complexes are essential for maintaining cell morphology, regulating cellular tension, and providing mechanical support to tissues. The downregulation of the tight junction and focal adhesion pathways suggests that intercellular connections and cellular support in the AK of the rumen tissue were compromised (Wang et al., 2021; Xue et al., 2021). A study by Meissner et al. (2017) demonstrated that prolonged low pH and VFA accumulation lead to dysfunction of rumen epithelial tight junctions, resulting in cell damage and reduced expression of intercellular tight junction proteins, which aligns with our findings. Moreover, the downregulation of the focal adhesion pathway further supports the view that low pH disrupts cellular connections and tissue structure in the rumen epithelium. We also observed a downregulation of the key endocytosis pathway, which regulates the absorption and uptake of proteins, signaling molecules, and other substances from the external environment (Pathak et al., 2023). Besides, the downregulated expression of genes in endocytosis pathway suggests the ability of ruminal AK tissue to absorb nutrients and other molecules was impaired by the overly low pH condition induced by high grain diet, which has been reported by others (Bull et al., 1965; Kern et al., 2016). In the modules significantly correlated with rumen papillae length, the enrichment of adhesion junction, and endocytosis pathways further supports that the ability of the rumen to acquire nutrients, signaling molecules, and extracellular substances is impaired.

An overly reduction in rumen pH leads to an enhanced exchange of extracellular VFA anions with intracellular ${\text{HCO}}_{3}^{-}$, resulting in intracellular acidification and initiating inflammation such as cell swelling (Meissner et al., 2017). Induced acute RA increases trans-epithelial electrical conductance and fluorescein permeability, with these effects persisting even after pH levels return to normal (Gozho et al., 2005; Klevenhusen et al., 2013). Zhang et al., (2016) reported that genes associated with rumen epithelial inflammation were significantly upregulated in cows fed high-grain diets. Along with the reduced thickness of the stratum corneum in AK of the rumen tissues, we also observed that genes related to the IL-17 signaling pathway were significantly enriched and upregulated in the MEgreen module. Activation of the IL-17 signaling pathway can promote the recruitment and activation of inflammatory cells, leading to an increased production of inflammatory mediators (Amatya et al., 2017). The enrichment of this pathway indicates the inflammatory state of the rumen tissue, suggesting that the rumen tissue AK extent is related to inflammation.

Differential expression analysis and weighted gene co-expression network analysis capture only a subset of the transcriptional features of the rumen (Botía et al., 2017). Gene set enrichment analysis evaluates the expression of all genes within specific functional pathways (Shi and Walker, 2007), while gseKEGG pathway similarity interaction network analysis reveals the synergistic interactions between these pathways (Kerseviciute and Gordevicius, 2023). Key abnormally expressed genes related to ruminal AK were identified by integrating the results from DEA, WGCNA, and GESA. *ACTR2* regulates the expression of Actin Related Protein 2, which is an important component of the cytoskeleton (Hobbs et al., 2019), is downregulated when the rumen environment acidifies, leading to disruption of tight junction and decreased cell barrier function (Liu et al., 2013). This allows pathogens to cross the barrier more easily (Zhao et al., 2018), triggering upregulation of *MMP9* in the IL-17 signaling pathway, which promotes the migration and infiltration of inflammatory cells, thereby enhancing the inflammatory response (Xie et al., 2015; Ma et al., 2021). Additionally, *MMP9* upregulation facilitates the formation of neutrophil extracellular traps (NETs) to capture and kill pathogens, although excessive NETs aggregation could lead to tissue damage (Leal et al., 2025). Furthermore, the upregulation of *ITGB7* promotes the recruitment of immune cells to the rumen epithelium, enhancing the local immune response (Koch et al., 2023). The disruption of intercellular connections and supporting structures in AK of the rumen tissue results in localized inflammation and an enhanced immune response, downregulating genes such as *SLC38A2* and *RAB5B*, which are involved in nutrient uptake and signaling molecule capture (Lv et al., 2022), and especially weakening the absorption of proteins. The qRT-PCR expression of key genes *ACTR2*, *MMP9*, *ITGB7*, and *SLC38A2* was consistent with the transcriptomic expression trend. This confirmed impaired tight junctions and an upregulated IL-17 signaling pathway in AK areas of the rumen, leading to downregulated protein digestion and absorption pathway.

This study focused specifically on local transcriptomic changes within the rumen epithelium to understand the molecular mechanisms of abnormal keratinization at the tissue level. However, systemic inflammatory markers and blood parameters would provide important insights into the broader physiological impacts of rumen AK. Future studies should incorporate blood sampling to measure circulating inflammatory cytokines, acute-phase proteins, and metabolic indicators to establish the systemic consequences of localized rumen epithelial damage.

## Conclusion

In the current study, IAKR was observed in several finishing lambs fed a barley-rich diet, and individuals with these lesions exhibited lower rumen pH. By conducting a comprehensive analysis of transcriptome data using DEA, WGCNA, and GSEA, and by verifying the results using qRT-PCR, we found that at the mRNA level, the keratinization abnormalities were associated with impaired tight junctions and upregulated IL-17 signaling. Further research is warranted to understand the long-term effects of AK on animal health and performance and to find out why individual animals develop AK while others do not.

## Supplementary Material

skaf352_Supplementary_Data

## Data and Model Availability Statement

The datasets generated and analysed during the current study are available in NCBI’s Gene Expression Omnibus (project number PRJNA1236648). The Supplementary Material for this article can be found online at: https://github.com/ztx2023/AK-rumen

## Abbreviations:

AK: abnormal keratinization area of the rumen
DEA: Differential Expression Analysis
DEGs: Differential expressed genes
FPKM: Fragments per kilobase of exon model per million mapped fragments
GSEA: Gene Set Enrichment Analysis
IAKR: individuals with abnormal keratinization rumen
INR: individuals with normal rumen
KEGG: Kyoto Encyclopedia of Genes and Genomes
MN: morphologically normal area of the rumen
NETs: neutrophil extracellular traps
PBS: Phosphate-buffered saline
PFA: Paraformaldehyde
RA: Rumen acidosis
SARA: Subacute rumen acidosis
VFA: volatile fatty acid
WGCNA: Weighted Gene Co-expression Network Analysis
